# Resolution of paraneoplastic hypoglycemia following nephroureterectomy for treatment of canine renal cell carcinoma: Case report

**DOI:** 10.3389/fvets.2023.1134098

**Published:** 2023-03-31

**Authors:** Yael Huerta, Jennifer Lenz, Brian Flesner, Brittany Abrams, Hayley Amerman, Maureen Griffin

**Affiliations:** ^1^Department of Clinical Sciences and Advanced Medicine, University of Pennsylvania School of Veterinary Medicine, Philadelphia, PA, United States; ^2^Department of Pathobiology, University of Pennsylvania School of Veterinary Medicine, Philadelphia, PA, United States

**Keywords:** renal cell carcinoma, paraneoplastic hypoglycemia, non-islet cell tumor hypoglycemia, nephroureterectomy, dog

## Abstract

**Objectives:**

To describe the clinicopathologic findings, imaging results, surgical treatment, and outcome of a dog with renal cell carcinoma (RCC) and paraneoplastic hypoglycemia.

**Animals:**

A 13-year-old female spayed mixed breed dog that was presented for facial twitching and neurologic decline and diagnosed with a renal mass and paraneoplastic hypoglycemia.

**Study design:**

Case report.

**Methods:**

Serum chemistry revealed severe hypoglycemia and normal renal values. Abdominal ultrasonography showed a large, heterogeneous, cavitated mass associated with the left kidney and no evidence of abdominal metastatic disease. Thoracic radiographs revealed no evidence of pulmonary metastatic disease. Fasted serum insulin was low concurrently with severe hypoglycemia. No other causes of hypoglycemia were detected, and paraneoplastic hypoglycemia was suspected.

**Results:**

After initial medical management of the dog's hypoglycemia, left nephroureterectomy was performed. Histopathology was consistent with RCC. Postoperatively, the dog's hypoglycemia resolved, and supplementation was discontinued. The dog remained stable and was discharged from the hospital 3 days after surgery. At 2-week, 3-month, and 5-month follow up evaluations, the dog remained euglycemic, and no definitive evidence of disease progression was detected. Eight months postoperatively, the dog was euthanized due to decline in mobility. Necropsy and histopathology revealed cerebral and spinal cord multifocal myelin sheath dilation and two primary pulmonary carcinomas with no evidence of recurrence or metastasis of the RCC.

**Conclusion:**

Surgical treatment of RCC with subsequent resolution of paraneoplastic hypoglycemia has not previously been reported in veterinary medicine. In this dog, nephroureterectomy for RCC resulted in immediate and sustained resolution of paraneoplastic hypoglycemia.

## Introduction

Paraneoplastic syndromes are clinical manifestations associated with an underlying cancer due to tumor secretion of hormones, peptides, or cytokines, or from immune cross-reactivity between malignant and normal tissues (1). Insulinoma, the most common neoplasm associated with hypoglycemia in dogs, is a functional tumor of beta cells (pancreatic islet cells) that secrete insulin independent of glucose levels, resulting in hypoglycemia. The pathophysiology of non-islet cell tumor hypoglycemia (NICTH) is predominantly associated with tumor production of the pro-hormones insulin-like growth factor I (IGF-1) and insulin-like growth factor II (IGF-2), which have a biologic activity very similar to that of insulin without concurrent elevations of serum insulin levels (2, 3).

NICTH has been rarely reported in dogs in association with melanoma, hemangiosarcoma, hepatoma, hepatocellular carcinoma, leiomyoma, leiomyosarcoma, mammary carcinoma, and renal carcinoma (4–10).Very few cases of documented paraneoplastic hypoglycemia associated with renal carcinoma exist in both human and veterinary literature (9, 11–17). Only one report of suspected paraneoplastic hypoglycemia in a dog with renal carcinoma exists (9). This dog had a renal mass and pulmonary nodules on staging evaluation. Medical treatment with steroids was attempted but was ultimately ineffective in this case, and euthanasia was elected. Necropsy findings were consistent with renal adenocarcinoma with pulmonary metastasis and no other significant lesions. Paraneoplastic hypoglycemia was presumed as a diagnosis of exclusion, and supportive care did not improve the dog's hypoglycemia. In humans with renal carcinoma and paraneoplastic hypoglycemia, several case reports state hypoglycemic resolution following nephrectomy, though this has never been demonstrated in a veterinary patient (11–13, 16).

Paraneoplastic hypoglycemia associated with renal carcinoma is rare, and to date no reports of treatment *via* nephrectomy and documentation of subsequent outcomes exist in veterinary medicine. The objective of this report is to describe the diagnostic techniques, surgical treatment, and outcome of a dog with renal cell carcinoma (RCC) and associated paraneoplastic hypoglycemia that underwent nephroureterectomy.

## Case description

A 13-year-old female spayed mixed breed dog was presented for neurologic signs. The owners previously noted facial tremors (suspected focal seizures) that resolved spontaneously 3 months prior to this visit. On the day of presentation, these clinical signs were noted again but progressed in severity, and the dog had collapsed several times at home. The owners also noted recent circling, altered behaviors, hyporexia, weight loss, and polyuria/polydipsia. The dog also had a history of two mammary carcinomas (grade 1 and grade 2) previously excised with complete histologic margins 2 years prior to this visit, with no evidence of metastasis prior to surgery or on subsequent restaging every 6 months. In addition, the dog had a long-term history of abnormal gait and intermittent loose stool. The dog was receiving oral carprofen (2.2 mg/kg twice daily) (Zoetis, Kalamazoo, MI), gabapentin (9 mg/kg twice daily) (Ascend Laboratories, Parsippany, NJ), and metronidazole (11 mg/kg twice daily) (Unichem Pharmaceuticals, East Brunswick, NJ) at the time of presentation.

On physical examination, the dog's vital parameters were within normal limits. A large, firm mass was palpated in the left, dorsal, mid-abdomen. The dog had an obtunded mentation, moderate pelvic limb ataxia, generally stiff gait, and diffuse epaxial and hind limb muscle atrophy. Two small (<1 cm) nodules associated with the right first and left third mammary glands were also noted.

Hematology revealed a mild non-regenerative anemia (HCT 36%; RR: 41–58), mild neutrophilia (11,080/μl; RR: 2,700–9,400), and mild lymphopenia (600/μl; RR: 900–4,700). Serum biochemistry revealed severe hypoglycemia (29 mg/dl; RR: 80–112). Renal values were within normal limits [creatinine 0.9 mg/dl (RR: 0.7–1.8), blood urea nitrogen (BUN) 11 mg/dl (RR: 5–30 mg/dl)], and all liver enzymes and pseudofunction parameters were within normal limits (aside from glucose). A fasted serum blood insulin-glucose panel and serum IGF-1 level were submitted. Serum insulin was decreased (4.3 μIU/ml; RR: 8.1–31.9) with concurrent severe hypoglycemia (12 mg/dl; RR: 81–118), and serum IGF-1 was within normal limits but at the low end of the reference range (5 nmol/l; RR: 4–95). Urinalysis revealed a urine specific gravity of 1.026 with 1+ protein, 3+ blood, and 0–1 white blood cells/high-power field.

**Figure 1:**
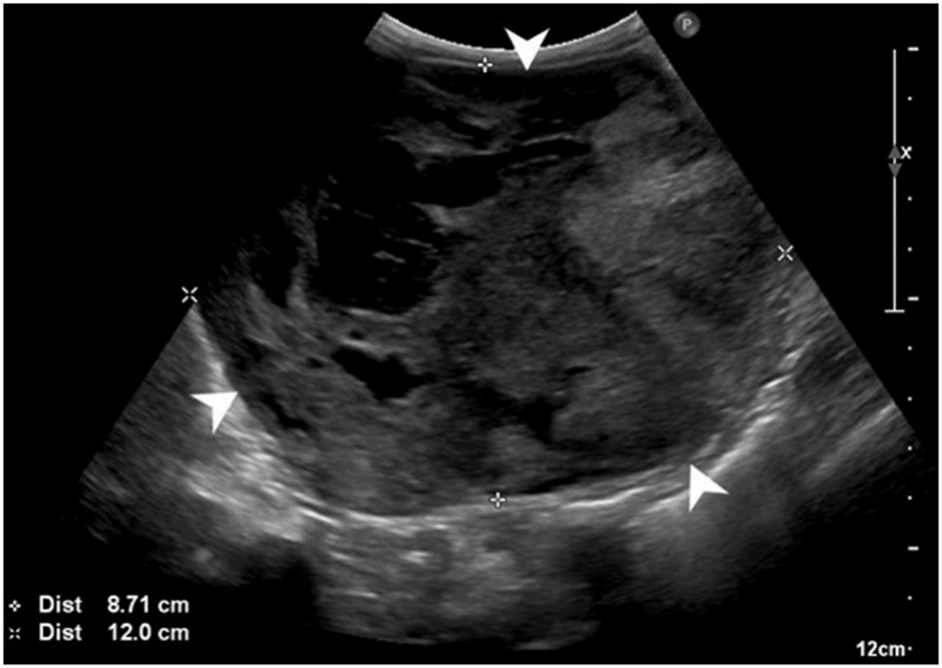
Preoperative abdominal ultrasonographic study. Large, cavitated, heterogeneous mass (white arrowheads) arising from the left kidney.

Abdominal ultrasonography showed a large (at least 8.7 × 12 cm), heterogeneous, cavitated mass extending cranially from the cranial pole of the left kidney and causing overall caudal displacement of the left kidney (Figure 1), as well as mild left renal lymphadenopathy (6.7 mm), diffuse peritoneal steatitis, and mild peritoneal effusion. Moderate bilateral degenerative renal changes with pyelectasia (left worse than right) were noted. No other significant abnormalities were detected. Thoracic radiographs revealed sternal lymphadenopathy, a bronchointerstitial pattern, caudal mediastinal widening, and multifocal disc space narrowing. There was no evidence of pulmonary metastatic disease.

Following detection of hypoglycemia, the dog was administered an IV bolus of dextrose 50% (0.29 g/kg) (Nova-Tech, Grand Island, NE) followed by IV isotonic crystalloid fluid therapy (80 ml/kg/day) (Plasma-Lyte A, Baxter Healthcare Corp., Deerfield, IL) with dextrose supplementation at 2.5%. The blood glucose (BG) was monitored closely every 2–4 h using an AlphaTrack glucometer (Zoetis, Parsippany-Troy Hills, NJ). The IV dextrose supplementation was progressively increased to 15% over the following 36 h due to persistent hypoglycemia. Constant rate infusions of glucagon (7 ng/kg/min) (Fresenius Kabi, Lake Zurich, IL) and dexmedetomidine (1 μg/kg/h) (Zoetis, Kalamazoo, MI) were also initiated 12 h after beginning medical management due to persistent hypoglycemia. Frequent feeding was also performed, and the dog's hypoglycemia resolved with these supportive therapies in hospital.

Despite resolution of the hypoglycemia, the dog's obtundation and mild ataxia persisted. After consultation with a board-certified veterinary neurologist, a forebrain lesion was suspected. The dog underwent general anesthesia, and magnetic resonance imaging (MRI) of the brain [GE HealthCare, 1.5 Tesla, Signa Explorer; sequences including sagittal and transverse T2w, transverse T2-FLAIR and T2^*^GRE, DWI/ADC, and transverse, dorsal, and sagittal T1w (pre- and post-contrast)] revealed a small interthalamic adhesion (~2.7 mm in height) with an irregularly triangular shape, mild bilaterally symmetrical periventricular T2w/T2-FLAIR hyperintensities (most pronounced along the caudal dorsolateral aspect of the lateral ventricles), no abnormal contrast enhancement, and moderate generalized ventriculomegaly, widened sulci, and thinning of the cortical gray matter throughout the cerebrum. These findings were consistent with generalized age-related brain atrophy and leukoaraiosis associated with canine cognitive dysfunction syndrome. No evidence of primary or metastatic neoplasia was detected.

**Figure 2:**
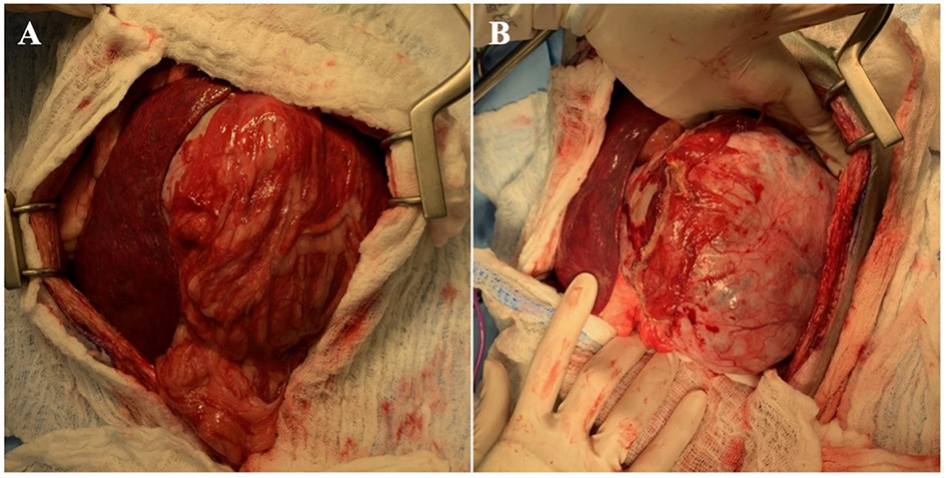
**(A)** Initial abdominal explore findings with left renal mass surrounded by omentum (right) and spleen (left). **(B)** Left renal mass following dissection of omental adhesions.

The dog subsequently underwent exploratory celiotomy to excise the renal mass. Abdominal explore revealed a large left renal mass (Figure 2) and mild generalized rounding and mottling of the liver. No overt pancreatic masses, enlarged lymph nodes, or other abnormalities were detected. Routine left nephroureterectomy was performed. An incisional biopsy of the left lateral liver was obtained *via* a suture-fracture technique. Gloves and instruments were changed, and the abdominal wall was closed routinely. Wide excision of the mammary nodules was subsequently performed, followed by routine subcutaneous and skin closure. No intraoperative complications occurred, and the dog recovered from anesthesia uneventfully.

**Figure 3:**
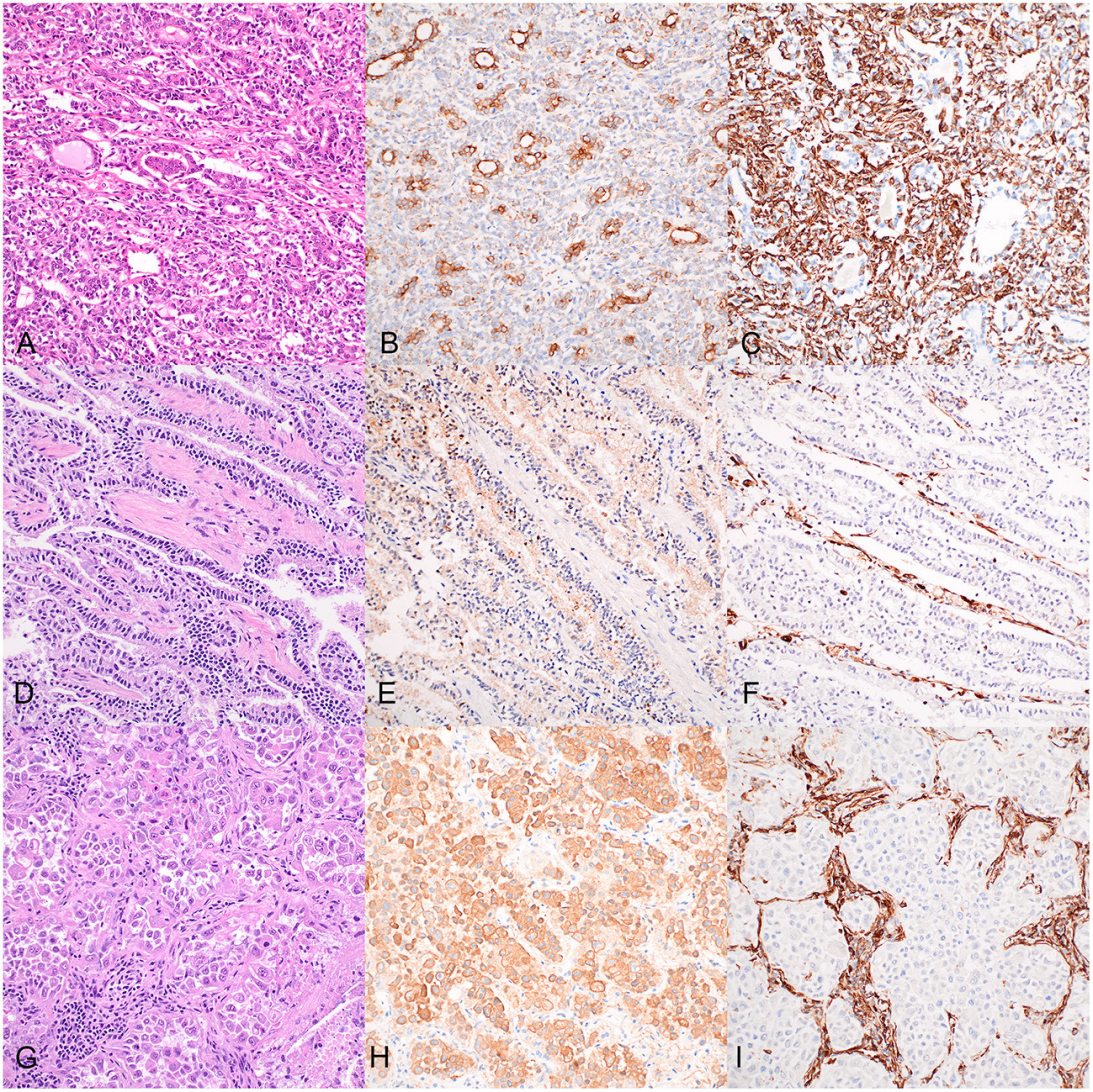
Histopathology and immunostaining of the dog's renal cell carcinoma **(A–C)**, accessory lung lobe neoplasm **(D–F)**, and right caudal lung lobe neoplasm **(G–I)**. 20X. **(A)** Renal cell carcinoma in which neoplastic epithelial cells are arranged in tubules, nests, and streams; hematoxylin and eosin (HE). **(B)** Cytokeratin immunohistochemistry (IHC) of the renal cell carcinoma: positive cytoplasmic staining of neoplastic epithelial cells. **(C)** Vimentin IHC of the renal cell carcinoma: positive cytoplasmic staining of neoplastic epithelial cells. **(D)** Accessory lung lobe neoplasm in which neoplastic cells are arranged in lepidic cords and papillary projections; HE. **(E)** Cytokeratin IHC of the accessory lung lobe neoplasm: moderate positive cytoplasmic labeling of neoplastic cells. **(F)** Vimentin IHC of the accessory lung lobe neoplasm: negative staining of the neoplastic cells. **(G)** Right caudal lung lobe neoplasm in which neoplastic cells are arranged in nests amongst abundant inflammation and necrosis; HE. **(H)** Cytokeratin IHC of the right caudal lung lobe neoplasm: strong positive cytoplasmic labeling of the neoplastic cells. **(I)** Vimentin IHC of the right caudal lung lobe neoplasm: negative staining of the neoplastic cells.

Histopathology of the dog's renal mass was consistent with RCC with a large central focus of cell dropout and necrosis in which the cells were arranged in tubules, nests, and streams and demonstrated moderate nuclear pleomorphism, no vascular invasion, and a mitotic count of 18 per ten 400X high-power fields (Figure 3A). Histopathology of the liver sample revealed no significant lesions. Histopathology of the mammary nodules were consistent with lobular hyperplasia and epitheliosis (right first mammary gland) and intraductal papillary adenoma (left third mammary gland); the mammary lesions were completely excised.

Postoperative therapy included methadone (0.1 mg/kg IV every 6 h) (Akorn, Lake Forest, IL) for the first 12 h, gabapentin (10 mg/kg orally every 8 h), trazodone (4.5 mg/kg orally every 8 h as needed) (NuCare Pharmaceuticals, Orange, CA), and IV isotonic crystalloid fluid therapy (60 ml/kg/day) (Plasma-Lyte A, Baxter Healthcare Corp., Deerfield, IL). Cardiovascular parameters and BG were closely monitored. The dog remained cardiovascularly stable, and BG was within normal limits without any supplementation immediately after surgery and throughout the duration of hospitalization. One day postoperatively, soft crackles were auscultated in all lung fields, though respiratory rate and effort were normal. Thoracic radiographs were performed and showed a mild cranioventral interstitial to alveolar pattern. Early bronchopneumonia or atelectasis were considered as differentials. Complete blood count revealed a mature neutrophilia (10,240/μl). Piperacillin/tazobactam (75 mg/kg IV every 6 h) (Sandoz, Princeton, NJ) was initiated to treat possible pneumonia and continued for 2 days, at which time antibiotics were discontinued due to persistent eupnea and normal auscultation.

The dog's mentation and overall neurologic status were noted to improve over the first 24 h after surgery. Serum chemistry panel 1 day after surgery revealed a mild elevation in creatinine (1.4 mg/dl) compared to preoperatively with normal BUN (17 mg/dl). IV fluid therapy was continued for an additional 36 h for renal support. Repeat chemistry panels every 24 h showed stable creatinine (1.1 and 1.3 mg/dl, respectively). The dog showed no interest in food the first day postoperatively, though no vomiting, nausea, or regurgitation were noted. Capromorelin (3 mg/kg orally every 24 h) (Elanco US, Greenfield, IN) was administered and the dog started eating 42 h after surgery. Three days postoperatively, the dog appeared comfortable and was stable with a good appetite, water intake, and activity level. The dog was discharged with the following medications: gabapentin (10 mg/kg orally every 8–12 h), trazodone (2.5 mg/kg orally every 8–12 h) as needed, and capromorelin (3 mg/kg orally every 24 h) as needed. An antioxidant-rich diet (Purina Pro Plan Bright Minds, St. Louis, MO) was offered for potential management of the dog's cognitive dysfunction.

The dog was presented for recheck evaluation 2 weeks postoperatively. The owner reported that the dog was active, interactive, and eating and drinking normally. No neurologic or respiratory signs had been noted at home. On physical examination, no pain was elicited on abdominal palpation, the ventral midline incision appeared healed, and neurologic examination was normal, including an alert and responsive mentation. A chemistry panel revealed stable creatinine (1.3 mg/dl) and slightly elevated BUN (32 mg/dl). The dog's BG was 91 mg/dl, and serum IGF-1 remained within normal limits (34 nmol/l), though increased relative to initial IGF-1 level.

At a 3 month follow up examination, the owner reported that the dog had been active and alert at home, though occasional circling was noted. On physical examination, the dog had a normal mentation and no neurologic deficits. Thoracic radiographs revealed a moderate to marked, diffuse bronchointerstitial pattern and a small soft tissue opacity nodule on the left lateral view suspected to represent an end on vessel or fluid-filled bronchus, though a primary pulmonary mass or pulmonary metastatic disease could not be ruled out. Abdominal ultrasonography revealed no evidence of local recurrence or metastatic disease and the previously identified enlarged left renal lymph node was not visible in this study.

At a 5 month follow up evaluation, the dog continued to be active, eating and drinking normally, and urinating normally. Physical examination revealed mild pelvic limb ataxia and circling to the left, suspected to be associated with the dog's progressive cognitive dysfunction. Hematology revealed a non-regenerative anemia (HCT 32.7%) and moderate lymphopenia (510/μl). Serum biochemistry revealed no abnormalities with stable creatinine (1.2 mg/dl) and euglycemia (93 mg/dl). Thoracic radiographs revealed a similar moderate, diffuse bronchointerstitial pattern, and a progressive soft tissue to mineral opaque nodule was noted on the left lateral view. Possible origins of this nodule included the liver, right lung, or body wall and differentials included dystrophic mineralization of a neoplastic or benign lesion. Abdominal ultrasonography revealed no evidence of local recurrence or intra-abdominal metastasis.

Eight months postoperatively, the owners noted a substantial decline in mobility. No overt seizures or collapse episodes were observed, though the dog continued to have cognitive decline. No further diagnostics were pursued, the dog was euthanized, and necropsy was performed. Gross findings revealed a 1 cm nodule filled with opaque, viscous material in the right caudal lung lobe. The accessory lung lobe had a 2 × 1.5 × 1 cm soft, irregular, tan nodule. The right kidney was mottled with multifocal to coalescing depressions on the capsular surface and light tan streaks that radiated from the renal pelvis into the medulla on cut surface. The brain and remaining viscera were grossly unremarkable.

Histopathology of the right caudal lung lobe lesion was consistent with a malignant neoplasm, in which a moderately demarcated neoplastic proliferation of polygonal cells was arranged in lobules and nests amongst the pre-existing pulmonary stroma (Figure 3G). The cells had abundant eosinophilic cytoplasm and demonstrated moderate pleomorphism with large round nuclei. This neoplastic population was surrounded by abundant inflammation and cellular debris. The accessory lung lobe lesion was consistent with a pulmonary carcinoma in which cuboidal to columnar neoplastic epithelial cells were arranged in long anastomosing cords in a lepidic pattern and papillary projections supported by a fine fibrovascular stroma (Figure 3D). These neoplastic cells had a moderate amount of finely granular, eosinophilic cytoplasm with occasional apical microvilli, and round centrally to basally located nuclei with mild pleomorphism and rare mitotic activity. The right kidney had moderate chronic lymphoplasmacytic pyelonephritis with interstitial fibrosis, segmental to global glomerulosclerosis, and mild multifocal chronic lymphoplasmacytic tubulointerstitial nephritis. Histopathology of the brain revealed mild multifocal myelin sheath dilation. The spinal cord had mild to moderate multifocal dilated myelin sheaths with myelinomacrophages and swollen axons (consistent with Wallerian degeneration). The liver demonstrated moderate centrilobular hepatocellular clearing and swelling (steroid hepatopathy).

Immunohistochemistry was performed for the pulmonary lesions to further characterize the cell of origin and whether they represented primary pulmonary neoplasms or metastatic disease from the previously diagnosed RCC (Figure 3). The RCC and both lung neoplasms were stained with antibodies against wide-spectrum cytokeratin (WSCK), vimentin, and thyroid transcription factor-1 (TTF-1). Each neoplasm stained negatively with TTF-1 (not shown). Approximately 60% of the neoplastic cells of the RCC demonstrated moderate to strong cytoplasmic labeling for WSCK (Figure 3B), and ~80% of RCC neoplastic cells demonstrated strong cytoplasmic labeling for vimentin (Figure 3C). These results support the diagnosis of RCC. In the neoplasm from the accessory lung lobe, there was moderate to strong cytoplasmic labeling in >90% of the neoplastic cells for WSCK (Figure 3E) and the vimentin stain was negative for the neoplastic cells (Figure 3F). These results, together with the classic histomorphology of this neoplasm, support a diagnosis of a primary pulmonary carcinoma (18). In the neoplasm from the right caudal lung lobe, there was diffuse strong cytoplasmic labeling of the neoplastic cells for WSCK (Figure 3H) and negative staining of the neoplastic cells with vimentin (Figure 3I). This similarly supports the suspicion of primary pulmonary carcinoma, despite the atypical histomorphologic features observed.

## Discussion

This case represents the first report of nephroureterectomy for treatment of RCC and associated paraneoplastic hypoglycemia in a dog, with documented sustained resolution of the dog's hypoglycemia postoperatively. Although a mechanism for the dog's hypoglycemia was not definitively determined, other causes of hypoglycemia, including elevated serum insulin level, sepsis/infection, and liver dysfunction were ruled out *via* bloodwork. The dog had no known toxin exposure, and even though a resting cortisol level was not obtained, the dog's electrolytes were normal, and the hypoglycemia resolved following nephrectomy without any treatment for hypoadrenocorticism, making this differential very unlikely. Thus, in this case, a paraneoplastic source of the dog's hypoglycemia is presumed as a diagnosis of exclusion. Although benign tumors can result in functional changes, mammary adenomas have not previously been reported in association with paraneoplastic hypoglycemia in any species (9, 11–17). Therefore, RCC was deemed to be the source of paraneoplastic hypoglycemia in this dog. As reported in several human patients with paraneoplastic hypoglycemia in association with RCC, immediate and long-term resolution of hypoglycemia was achieved after the renal mass was excised *via* nephrectomy (11–13, 16).

NICTH has been documented in association with tumor production of IGF-1 and IGF-2, with IGF-2 elevations generally suspected to be the predominant mechanism (2, 3). In considering the etiology of RCC-associated hypoglycemia in this dog, pre- and post-operative IGF-1 serum values were measured. The dog's serum IGF-1 level was bordering the low end of the reference range preoperatively and in the mid-reference range postoperatively, such that tumor production of IGF-1 is considered unlikely to be the source of paraneoplastic hypoglycemia for this case. This is consistent with findings of a previous study using highly sensitive and specific radioimmunoassays in humans, in which patients with NICTH commonly had low serum IGF-1 levels and normal to increased serum IGF-2 levels (19). IGF-2 produced by RCC is a possible source of this dog's paraneoplastic hypoglycemia, with associated relatively reduced IGF-1 levels preoperatively. However, no laboratory quantification assays for serum IGF-2 are currently available in dogs, and other potential etiologies remain possible.

Unilateral renal neoplasia is a well-documented indication for nephroureterectomy in dogs (20). In this case of localized, non-metastatic neoplasia effacing a portion of the kidney and suspected to be causing paraneoplastic hypoglycemia, complete nephroureterectomy was deemed indicated. Although the dog's renal values were normal on serum chemistry, degenerative changes of the renal parenchyma bilaterally were noted on ultrasonography, and the risk of renal dysfunction postoperatively was discussed with the owners. Nevertheless, due to the strong indication for nephrectomy in this case, proceeding with surgery without further evaluation of the contralateral renal function with dynamic imaging was deemed appropriate. However, functional evaluation of the remaining kidney prior to nephrectomy can be considered with modalities such as dynamic renal scintigraphy in dogs (20). Postoperative monitoring of renal values and renal supportive therapy should be performed as standard of care following nephrectomy. The dog of this report maintained appropriate renal function postoperatively. The current report describes a good surgical outcome following nephrectomy with long-term glycemic and tumor control and no evidence of recurrent or metastatic RCC at the time of euthanasia 8 months postoperatively.

Limitations of the current report include the retrospective nature of data collection and lack of definitive mechanism identification for the paraneoplastic hypoglycemia associated with the dog's RCC. Internal testing of previously published antibodies failed to demonstrate reliable, specific binding to canine positive control tissues, suggesting a lack of cross-reactivity with canine IGF-1 and IGF-2 that subsequently precluded our ability to evaluate tumor expression of IGF-1 and IGF-2 *via* IHC (21, 22). Further, while NICTH secondary to the benign mammary nodules concurrently removed at the time of nephrectomy is considered less likely, rare descriptions of NICTH in human patients with benign phyllodes tumors of the breast have been reported (23). Phyllodes tumors arise from the periductal stromal cells of the breast and are recognized in dogs, but they have distinct morphological and histologic features not seen in this case (24). In addition, concurrent cognitive dysfunction is a confounding factor in this dog's case which made it difficult to evaluate neurologic signs in association with glycemic status, though the dog's neurologic signs improved and hypoglycemia resolved postoperatively.

In conclusion, findings of this case support that nephroureterectomy can result in immediate and long-term resolution of paraneoplastic hypoglycemia associated with RCC in dogs. Thorough diagnostic evaluation should be pursued to rule out other potential causes of hypoglycemia before considering surgery. In the absence of significant contralateral renal compromise and metastatic disease upon preoperative staging, nephroureterectomy should be considered the treatment of choice for dogs with a renal mass and suspected paraneoplastic hypoglycemia. Evaluation of a larger cohort of dogs with RCC and NICTH is required to further assess long-term outcomes following nephrectomy, and additional testing is needed to determine the mechanism of paraneoplastic hypoglycemia in dogs with RCC.

## Data availability statement

The original contributions presented in the study are included in the article/supplementary material. Further inquiries can be directed to the corresponding author.

## Ethics statement

Ethical review and approval was not required for the study because this is a retrospective case report. All diagnostics and treatments were approved by the owners. Written informed consent was obtained from the owners of this animal for the publication of this case report.

## Author contributions

MG, BA, JL, and BF participated in the diagnosis, treatment, and management of the dog in this report. HA participated in the diagnosis and histopathological evaluation of the dog in this report. All authors participated in the data acquisition and manuscript preparation. All authors contributed to the article and approved the submitted version.
